# Preconceptional Maternal Vegetable Intake and Paternal Smoking Are Associated with Pre-implantation Embryo Quality

**DOI:** 10.1007/s43032-020-00220-8

**Published:** 2020-06-15

**Authors:** Jeffrey Hoek, Sam Schoenmakers, Esther B. Baart, Maria P. H. Koster, Sten P. Willemsen, Eva S. van Marion, Eric A. P. Steegers, Joop S. E. Laven, Régine P. M. Steegers-Theunissen

**Affiliations:** 1 Department of Obstetrics and Gynecology, Erasmus MC, University Medical Center, Dr. Molewaterplein 40, 3015 GD Rotterdam, the Netherlands; 2 Department of Biostatistics, Erasmus MC, University Medical Center, Rotterdam, the Netherlands; 3 Division of Reproductive Endocrinology and Infertility, Department of Obstetrics and Gynecology, Erasmus University Medical Center, Rotterdam, the Netherlands

**Keywords:** Lifestyle, Vegetables, Assisted reproductive technology, Embryo development, Nutrition, Smoking

## Abstract

Inadequate nutrition and lifestyle behaviors, particularly during the periconception period, are associated with a negative impact on embryonic and subsequent fetal development. We investigated the associations between parental nutritional and lifestyle factors and pre-implantation embryo development. A total of 113 women and 41 partners, with a corresponding 490 embryos, who underwent intracytoplasmic sperm injection (ICSI) treatment subscribed to the mHealth coaching platform “Smarter Pregnancy.” At baseline, nutrition and lifestyle behaviors (intake of fruits, vegetables, folic acid, and smoking and alcohol use) were identified and risk scores were calculated. A lower risk score represents healthier behavior. As outcome measure, a time-lapse morphokinetic selection algorithm (KIDScore) was used to rank pre-implantation embryo quality on a scale from 1 (poor) to 5 (good) after being cultured in the Embryoscope™ time-lapse incubator until embryonic day 3. To study the association between the nutritional and lifestyle risk scores and the KIDScore in men and women, we used a proportional odds model. In women, the dietary risk score (DRS), a combination of the risk score of fruits, vegetables, and folic acid, was negatively associated with the KIDScore (OR 0.86 (95% CI 0.76 to 0.98), *p* = 0.02). This could mainly be attributed to an inadequate vegetable intake (OR 0.76 (95% CI 0.59 to 0.96), *p* = 0.02). In men, smoking was negatively associated with the KIDscore (OR 0.53 (95% CI 0.33 to 0.85), *p* < 0.01). We conclude that inadequate periconceptional maternal vegetable intake and paternal smoking significantly reduce the implantation potential of embryos after ICSI treatment. Identifying modifiable lifestyle risk factors can contribute to directed, personalized, and individual recommendations that can potentially increase the chance of a healthy pregnancy.

**Electronic supplementary material:**

The online version of this article (10.1007/s43032-020-00220-8) contains supplementary material, which is available to authorized users.

## Introduction

Subfertility is still an increasing problem in the Western world, which can be attributed to postponing pregnancy, but also to a decline in sperm count, increasing age of women at the time of conception, obesity, smoking, and other poor lifestyles [1–3]. Despite novel developments in assisted reproductive technology (ART), high rates of implantation failure and early pregnancy loss are still seen after the transfer of selected, morphologically high quality, pre-implantation embryos [4].

Pre-implantation embryo development can be studied using the EmbryoScope™, which incorporates a specialized built-in microscope designed for automated time-lapse embryo assessment by acquiring images [5]. The EmbryoScope™ provides a controlled culture environment and captures comprehensive information on embryo development without the need for handling or disturbing the developing embryo. The use of embryo morphokinetics by timing of embryo developmental events, available through continuous time-lapse monitoring, has added another dimension to current traditional morphology classification scores used to predict embryo implantation potential and viability [6, 7]. Animal studies have shown that single embryo developmental kinetics at the cleavage stage are reflective of culture conditions, but also of embryo metabolism, genetic integrity, and blastocyst formation and quality [8–11]. To assist in embryo selection as part of IVF treatment, the KIDScore algorithm was developed as a generally applicable morphokinetic algorithm suitable to rank day 3 embryos, originating from different culture conditions and fertilization methods. Embryos are ranked in five groups predicting their ability to develop into a blastocyst with an area under the curve (AUC) of 0.75 and implantation potential with an AUC of 0.65 (indicative of intermediate prediction) [12]. Interestingly, a recent study showed that the KIDScore was superior regarding predicting implantation and ongoing pregnancy rates when compared to only scoring embryo morphology [13].

Most reproductive challenges, such as fertility problems, miscarriages, congenital malformations, and fetal growth restriction, largely originate in the periconceptional period, which ranges from at least 14 weeks before conception until 10 weeks after conception [14–16]. Inadequate nutrition and lifestyle behaviors particularly during the periconception period are associated with a negative impact on the development of the embryo and subsequent fetal development [17]. Nutrition and lifestyle behaviors are specifically of clinical interest since these factors are modifiable. Couples contemplating pregnancy are often not aware of their inadequate nutrition and lifestyle behaviors and the detrimental effects on reproduction [18]. To investigate the effect of adherence to general dietary recommendations in couples undergoing IVF/ICSI treatment, our group studied the association with the chance of ongoing pregnancy. Improvement of adherence to the nutritional recommendations of the Dutch Nutrition Centre (covering the intake of six main food groups namely fruits, vegetables, meat, fish, whole wheat products, and fats) resulted in a 65% increase of ongoing pregnancy after IVF-ICSI treatment [19]. Furthermore, inadequate nutritional behaviors of the mother during pregnancy can also have detrimental consequences for the health of the offspring later in life, where earlier age of puberty-onset and a decline in ovarian follicle reserve have been reported [20]. Furthermore paternal obesity and nutritional factors are also linked to sperm quality and epigenetic profiles, possibly also affecting embryo quality and pregnancy outcomes [21, 22]. We hypothesize that paternal nutrition and lifestyle factors affect multiple pathways involved in the (patho)physiology of sperm quality, such as inflammation, vascular pathways, and epigenetics, that can also influence the development of pre-implantation embryos.

Nutrition and lifestyle behaviors can be easily assessed using the online mHealth program Smarter Pregnancy, which effectively improves the intake of fruits, vegetables, and folic acid supplements and stop smoking and use of alcoholic drinks [23]. The effect of nutrition and lifestyle behaviors on fertility and pregnancy outcomes are widely studied; however, their influence on pre-implantation embryo development is limited. Investigating pre-implantation embryo parameters in vitro provides a unique insight into the direct impact of maternal and paternal factors through oocyte and sperm, respectively, independent of the in vivo utero maternal environment. Therefore, the aim of this study is to investigate the associations between the five Smarter Pregnancy lifestyle behaviors (vegetables, fruit, folic acid, smoking, and alcohol use) of both men and women and the quality of development of pre-implantation embryos cultured in the EmbryoScope™ as a marker of implantation potential and assessed by the KIDScore algorithm.

## Materials and Methods

### Study Design, Population, and Patient Inclusion

In a prospective cohort study, couples that underwent ICSI treatment were included when embryos were cultured in the EmbryoScope™ time-lapse incubator and baseline data on the five nutrition and lifestyle behaviors of the mHealth program Smarter Pregnancy were available. Couples were included between October 2014 until December 2017 at the Erasmus MC, University Medical Center, Rotterdam, the Netherlands [23].

Patients had to be at least 18 years of age and had to have a good understanding of Dutch speaking and writing. After the introduction of the EmbryoScope™ time-lapse incubator in our clinic, it was mostly used for cycles from patients undergoing ICSI treatment, as in this situation, oocytes can be submitted to time-lapse culture directly after injection and resulting embryos can benefit optimally from undisturbed culture. Only in the more recent years, we also cultured some embryos from IVF treatment cycles in the EmbryoScope™. In this case, fertilized oocytes are submitted to time-lapse culture the day after insemination, after pronuclear inspection. In these cases, the time point of fertilization is less accurate than after ICSI and pronuclear appearance cannot be assessed. We therefore excluded these cycles for the current analysis. Furthermore, we excluded patients with no available data of the Smarter Pregnancy program.

### Ethical Approval

This study was conducted according to the guidelines laid down in the Declaration of Helsinki and all procedures involving patients were approved by the Medical Ethical Institutional Review Board of the Erasmus, University Medical Center, Rotterdam, the Netherlands. Written informed consent was obtained from all female and male participants at enrolment.

### In Vitro Fertilization Procedures

Ovarian stimulation, oocyte retrieval, the ICSI procedures, and assessment of embryo morphology were performed as described previously [24]. Inseminated oocytes were cultured in the EmbryoScope™ in Sage 1-step culture medium (Origio/Cooper Surgical™, Denmark) at 36.8 °C, 7% oxygen, and 5% carbon dioxide. Embryo evaluation and selection for transfer was carried out on day 3 after oocyte retrieval, where selection was based on developmental stage and morphology. Embryos were ranked according to the number of blastomeres, fragmentation, size equality, and signs of early compaction. Top-ranked embryos consisted of 8 equally sized blastomeres with no to little fragmentation. Supernumerary embryos were cultured until day 4, when selection for cryopreservation was performed based on the degree of embryo compaction and the presence of fragmentation.

### Time-Lapse Imaging and Analysis of Morphokinetic Parameters

**Fig. 1:**
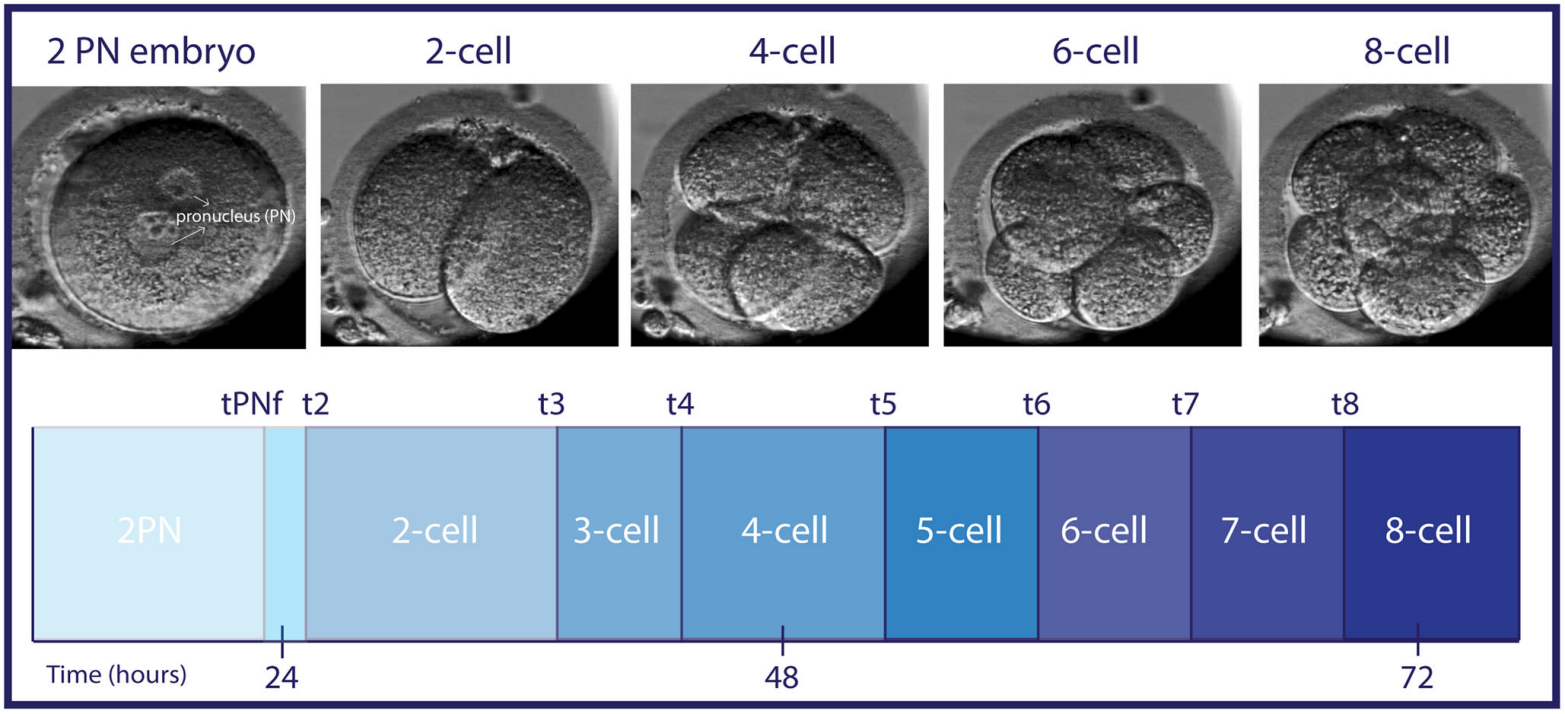
Embryo development from 1 cell to 8 cells (embryonic day 1 till day 3) as seen from the EmbryoScope™ with the corresponding time points used for the KIDScore

Embryo images were automatically recorded in seven focal planes (15-μm intervals, 1280 × 1024 pixels, 3 pixels per micrometer, monochrome CCD camera, single red LED 635-nm duration < 0.1 s per image, total light exposure time < 50 s per day per embryo) every 10 min until embryo day 3. Manual annotations were performed by specifically trained members of our team according to the definitions and guidelines by Ciray and colleagues [25]. Time of pronuclei appearance (tPNa) was defined as the appearance of both pronuclei, whereas the time of pronuclei fading (tPNf) was the first frame where both pronuclei were faded. The time points t2, t3, t4, t5, t6, t7 and t8 render the exact timing of reaching the 2, 3, 4, 5, 6, 7- and 8-cell stage of an individual embryo (Fig. 1). Team members performed a proficiency test to check consistency of annotations within and between observers. Extremely close agreement (ICC > 0.95) was observed for the pronuclear stage and the first cleavage divisions until the 5-cell stage, moderate agreement was observed for identifying the 6 to 8cell stage (ICC 0.23–0.40).

### Application of the KIDScore Algorithm

To assess embryo quality and the implantation potential of a pre-implantation embryo, the KIDScore was used [12]. The KIDScore ranges from 1 till 5 and is based on six annotations: the number of pronuclei equals 2 at the 1-cell stage, time from insemination to pronuclei fading (tPNf). Time to the 2-, 3-, or 5-cell stage (t2, t3, t5) and the number of cells 66 h after insemination (Fig. 1). The KIDScore is a deselection algorithm, where a decision tree with specific cut-off values determines which score is allocated to an individual embryo. Embryos classified as score 1 show a developmental pattern indicative of a low developmental and implantation potential (an observed average chance of implantation of 5%), whereas score 5 embryos follow a pattern indicative of a high potential (36%) [12].

### mHealth Program Smarter Pregnancy

The mHealth program Smarter Pregnancy (https://www.slimmerzwanger.nl; https://www.smarterpregnancy.co.uk) is a (cost) effective tool to improve nutrition and lifestyle [26]. Couples who wanted to participate in the program were subscribed to the mHealth program at the moment of fertility intake in the outpatient clinic of the Erasmus MC University Medical Center, Rotterdam. The Smarter Pregnancy program offers online coaching for a period of 6 months, focusing on four of the most prevalent inadequate behaviors, i.e., vegetables, fruits, alcohol, and folic acid supplement intake, and smoking as the behavior with the strongest detrimental effects on fertility and pregnancy outcome [17, 27–29]. The guidelines of the Netherlands Nutrition Centre were used to set the adequate daily intakes of at least 200 g of vegetables, two pieces of fruit, 400 μg of folic acid supplements, no smoking, and no use of alcohol. A baseline questionnaire was used to determine the presence of these five nutrition and lifestyle behaviors, which each were translated in a risk score. A high-risk score represents unhealthy nutrition or lifestyle. Intake of fruits, vegetables, folic acid supplements, and alcohol use was depicted on a scale from 0 to 3 and smoking was depicted on a scale from 0 to 6. Vegetable and fruit intake were both subdivided into a risk score of 0, 1.5, or 3, in which 0 represents an adequate daily intake (Table 1). The total risk score (TRS) was defined as the sum of all risks per behavior. The dietary risk score (DRS) is the combined total of fruits, vegetables, and folic acid supplement intake risk scores, while the lifestyle risk score (LRS) is the combined total of alcohol and smoking risk scores.

**Table 1:** Baseline characteristics of women and men in the entire study population

	Women (*N* = 113)	Missing	Men (*N* = 41)	Missing
Age, years	32.4 (29.1–35.1)	0	34.0 (29.5–41.5)	0
Ethnicity				
Western	95 (84%)	0	41 (100%)	
Non-Western	18 (16%)		0 (0%)	
Education				
Low	8 (7%)	0	3 (7%)	
Middle	47 (42%)		14 (34%)	
High	58 (51%)		24 (59%)	
BMI (measured), kg/m^2^	23.2 (21.7–26.0)	4	24.0 (22.6–27.5)	0
BMI category				
< 25	66 (59%)	1	24 (59%)	0
≥ 25	46 (41%)		17 (41%)	
Diagnosis category:		0		0
Only male factor	92 (81%)		32 (78%)	
Of which surgically retrieved sperm	51		17	
Only female factor	2 (2%)		1 (2%)	
Anovulation	2		1	
Tubal pathology	0		0	
Both male and female	17 (15%)		7 (18%)	
Of which surgically retrieved sperm	6		3	
Anovulation	16		6	
Tubal pathology	1		1	
Unexplained	2 (2%)		1 (2%)	
Time between activation Smarter Pregnancy and oocyte retrieval, days	49 (35–126)	0	45 (34.5–97)	0
Vegetable risk score				
0 (≥ 200 g/day)	31 (28%)	3	14 (34%)	0
1.5 (150–199 g/day)	22 (20%)		8 (20%)	
3 (< 150 g/day)	57 (52%)		19 (46%)	
Fruit risk score				
0 (≥ 2 pieces/day)	46 (42%)	4	18 (44%)	0
1.5 (1,5–1.9 pieces/day)	13 (12%)		8 (20%)	
3 (< 1.5 pieces/day)	50 (46%)		15 (36%)	
Folic acid risk score				
0 (0.4 mg/day)	111 (98%)	0	n/a	
3 (no usage)	2 (2%)			
Smoking risk score				
0 (no smoking)	107 (98%)	4	38 (93%)	0
1 (1–5 cigarettes/day)	0 (0%)		0 (0%)	
3 (6–14 cigarettes/day)	2 (2%)		2 (5%)	
6 (≥ 15 cigarettes/day)	0 (0%)		1 (2%)	
Alcohol risk score				
0 (no alcohol)	67 (61%)	4	13 (32%)	0
1.5 (1–2 units/day)	39 (36%)		24 (59%)	
3 (≥ 2 units/day)	3 (3%)		4 (9%)	

Data are presented as medians (IQR) or number of subjects (%). *IQR*, interquartile range; *BMI*, body mass index

### Study Parameters

Exposure variables as described above were extracted from the Smarter Pregnancy database. Electronic patient files were used to extract data on age and standardized anthropometric measurements carried out at intake, including maternal height with 0.1-cm accuracy and weight with 0.1-kg accuracy (anthropometric rod and weighing scale; SECA, Hamburg, Germany), as well as information about diagnosis of subfertility and oocyte retrieval date.

As outcome variable, we used the KIDScore as described above. In our hospital, the KIDScore is not used as a decision tool to select embryos for either transfer or cryopreservation. This decision is made by the embryologist based on a single morphological assessment at 66–68 h post fertilization. Only embryos with normal fertilization, as evidenced by the appearance of two pronuclei, that were subsequently transferred or cryopreserved were respectively annotated for research purposes.

### Statistical Analysis

Baseline characteristics of the female and male population in the current study are depicted as median or number with the corresponding interquartile range (IQR) or percentage. All analyses were performed using SPSS package 21.0 (IBM SPSS Statistics, Armonk, NY) and R (R: A language and Environment for Statistical Computing, version 3.1.3, 2015 for Windows, R Core Team, Vienna, Austria). To study the association between the nutritional and lifestyle risk scores and the KIDScore in men and women, we used a proportional odds model [30]. This is a model for ordinal outcomes like the KIDScore, using the ordinal package in R (Rune Haubo B Christensen). Challenging in pre-implantation analysis is the fact that couples usually have multiple embryos per cycle and normal regression analysis does not account for this clustering. Random subjects effects are used in the proportional odds model to account for this clustering. To adjust for potential confounders, two different models were constructed for the analysis. In the first model, no adjustments were made (crude model). The second model was adjusted for the covariate maternal age in the study population of women and for maternal age and for the risk score of the corresponding couples’ female risk score in the population of males. Subgroup analyses were performed in women with overweight/obesity (BMI ≥ 25 kg/m^2^) and normal weight (BMI < 25 kg/m^2^). The effect estimates of the models were transformed into odds ratios using the exponential function on the effect estimate. This odds ratio represents the chance of an individual embryo proceeding to a 1 point higher KIDScore given the associated maternal risk score. In the proportional odds model this odds ratio is assumed to be constant across all levels of the KIDScore.

To study the association between the fraction of discarded embryos and the nutrition and lifestyle risk scores, we used a generalized linear mixed model (GLMM) approach. Similar to the models discussed above, in the first model no adjustments were made (crude model) where the second model was additionally adjusted for the covariate maternal age. The effect estimates of the models were again transformed into odds ratios using the exponential function on the effect estimate. This odds ratio represents the chance of ICSI treatment resulting in a discarded embryo based on the individual risk score. Since nearly all women used folic acid supplements and nearly none were smokers analyzing these two behaviors was not possible.

## Results

**Fig. 2:**
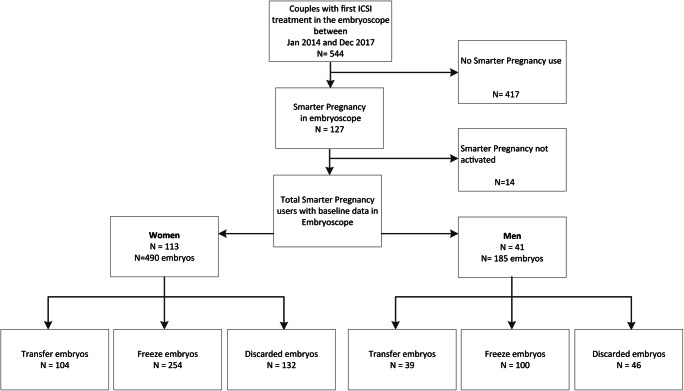
Flowchart of inclusions and exclusions of the study population

Between January 2014 and December 2017, embryos of 544 couples who underwent their first IVF/ICSI cycle were cultured in the EmbryoScope™. Of these couples, 417 (76.5%) were not subscribed to Smarter Pregnancy. In the end, 113 couples were included, of which 41 of the men also participated. In all included couples, fertilization was performed by using intracytoplasmic sperm injection (ICSI), which resulted in a total of 490 embryos that were cultured in the EmbryoScope™. Of these 490 embryos, 104 embryos were transferred, 254 were frozen, and 132 were of poor quality and thus discarded **(**Fig. 2**)**. From the 41 couples of which the male partner also participated, a total of 185 embryos were cultured in the EmbryoScope™. Of these 185 embryos (which is a subset of the total amount of 490 embryos), 39 embryos were transferred, 100 were frozen, and 46 were of poor quality and thus discarded **(**Fig. 2**)**.

### Women

The median age and BMI of the women was respectively 32.4 (IQR 29.1–35.1) years and 23.2 (IQR 21.7–26.0) kg/m^2^, of which 66 (59%) women had a normal BMI and 46 (41%) were overweight/obese (BMI >25 kg/m2). Most women were of Western origin (84%) and were highly educated (51%). The main reason for ICSI treatment was male factor subfertility (81%), of which in 55% of the cases sperm was retrieved surgically. Other indications included unexplained subfertility (2%), female factor subfertility (2%), and combined male-female factor subfertility (15%). The average time between completing the screening of the Smarter Pregnancy program and oocyte retrieval was 49 days (IQR 35–126 days). Most women had inadequate intake of vegetables (*n* = 79 (72%)) and fruits (*n* = 63 (58%)). Only two women did not take folic acid supplements (2%). The vast majority of all women did not smoke (*n* = 107 (98%)) and did not consume alcohol (*n* = 67 (61%)) **(**Table 1).

The results from the proportional odds model, indicative of the odds for an individual embryo getting a 1 point higher KIDScore given the individual risk scores, show that the vegetable risk score for the total population of women was negatively associated with the KIDScore with an effect estimate of − 0.28 and an odds ratio of 0.76 (95% CI 0.59 to 0.96) (Table 2a). After adjustment for maternal age, the effect remained statistically significant with an effect estimate of − 0.28 and an odds ratio of 0.76 (95% CI 0.59 to 0.96). No significant associations were observed for fruit intake and alcohol consumption. Furthermore, the DRS was also significantly associated with the KIDScore with an effect estimate of − 0.15 and a corresponding odds ratio of 0.86 (95% CI 0.76 to 0.98). Subgroup analysis showed that the effect of vegetable intake alone was only pronounced in overweight/obese women, with an effect estimate of − 0.55 and an odds ratio of 0.58 (95% CI 0.37 to 0.91). This effect even increased after adjustment for maternal age with an effect estimate of − 0.63 and an odds ratio of 0.53 (95% CI 0.32 to 0.87) (Table 2b). The associations between vegetable intake, fruit intake, and DRS and KIDScore are visually depicted in Supplemental figure **1****.** In women with a normal BMI, no significant associations were observed between the KIDScore and vegetables and fruit intake and alcohol consumption (Supplemental table **1**). When analyzing the risk of developing embryos with poor quality given the individual risk scores, the effect estimate for the TRS was 0.10 with a corresponding odds ratio of 1.11 (95% CI 0.92 to 1.33) after adjustment for maternal age (Supplemental table **2**), indicating that the TRS was not associated with the chance of a discarded embryo.

**Table 2:** Effect estimates and odds ratios from the proportional odds model for the nutrition and lifestyle risk scores on the KIDScore for (a) total study population of women, (b) overweight women only, and (c) total study population of males

	Effect estimate	Odds ratio (95% CI)	*P* value	Effect estimate	Odds ratio (95% CI)	*P* value
Total study population of women	Crude			Adjusted^*^		
Total risk score	− 0.13	0.88 (0.78 to 1.00)	0.049	− 0.13	0.88 (0.78 to 1.00)	0.047
Dietary risk score	− 0.15	0.86 (0.76 to 0.98)	0.025	− 0.15	0.86 (0.76 to 0.98)	0.024
Lifestyle risk score	0.15	1.16 (0.78 to 1.71)	0.47	0.07	1.08 (0.76 to 1.53)	0.42
Vegetable intake	− 0.28	0.76 (0.59 to 0.96)	0.03	− 0.28	0.76 (0.59 to 0.96)	0.02
Fruit intake	− 0.14	0.87 (0.68 to 1.11)	0.25	− 0.16	0.86 (0.70 to 1.05)	0.24
FA supplement use	n/a			n/a		
Alcohol use	0.15	1.16 (0.78 to 1.71)	0.62	0.07	1.08 (0.76 to 1.53)	0.68
Smoking	n/a			n/a		
Overweight women only	Crude			Adjusted^*^		
Total risk score	− 0.29	0.75 (0.57 to 0.98)	0.04	− 0.30	0.74 (0.56 to 0.98)	0.04
Dietary risk score	− 0.31	0.73 (0.56 to 0.97)	0.029	− 0.31	0.74 (0.55 to 0.98)	0.033
Lifestyle risk score	0.06	1.06 (0.52 to 2.19)	0.87	0.02	1.01 (0.47 to 2.17)	0.97
Vegetable intake	− 0.55	0.58 (0.37 to 0.91)	0.02	− 0.63	0.53 (0.32 to 0.87)	0.01
Fruit intake	− 0.18	0.84 (0.56 to 1.24)	0.37	− 0.18	0.84 (0.56 to 1.26)	0.39
FA supplement use	n/a			n/a		
Alcohol use	0.06	1.06 (0.52 to 2.19)	0.87	0.01	1.01 (0.47 to 2.17)	0.97
Smoking	n/a			n/a		
Total study population of males	Crude			Adjusted^#^		
Total risk score	− 0.13	0.88 (0.73 to 1.06)	0.18	− 0.11	0.90 (0.74 to 1.09)	0.28
Dietary risk score	− 0.08	0.92 (0.74 to 1.15)	0.45	− 0.03	0.97 (0.76 to 1.24)	0.83
Lifestyle risk score	− 0.23	0.80 (0.57 to 1.11)	0.18	− 0.27	0.76 (0.55 to 1.06)	0.11
Vegetable intake	− 0.21	0.81 (0.58 to 1.13)	0.22	− 0.13	0.88 (0.57 to 1.37)	0.57
Fruit intake	0.02	1.02 (0.74 to 1.42)	0.90	0.06	1.06 (0.76 to 1.48)	0.75
Alcohol use	0.14	1.15 (0.72 to 1.83)	0.57	0.08	1.08 (0.67 to 1.73)	0.75
Smoking	− 0.63	0.54 (0.34 to 0.85)	<0.01	− 0.63	0.53 (0.33 to 0.85)	<0.01

Crude model: no adjustments made

Adjusted model^*^: model 1 + adjusted for maternal age

Adjusted model^#^: model 1 + adjusted for maternal age and the corresponding risk score from the women

### Males

The median age and BMI of the male partner was respectively 34.0 (IQR 29.5–41.5) years and 24.0 (IQR 22.6–27.5). In males, 24 (59%) had a normal BMI (> 18.5 to < 25 kg/m^2^) and 17 (41%) were overweight. All men were of Western origin and most were highly educated (59%). The main indication for ICSI treatment was male factor subfertility (78%), of which in 53% of the cases sperm was retrieved surgically. The average time between completing the Smarter Pregnancy screening and oocyte retrieval was 45 days (IQR 34.5–97). The vast majority of men had an inadequate intake of vegetables (*n* = 27 (66%)) and fruits (*n* = 23 (56%)). A total of 38 (93%) were non-smokers, while refraining from alcohol was reported by only 13 men (32%) **(**Table 1**)**.

The results from the proportional odds model show that the risk score for smoking was negatively associated with the KIDScore with an effect estimate of − 0.63 and an odds ratio of 0.54 (95% CI 0.34 to 0.85) for the crude model and an effect estimate of − 0.63 and an odds ratio of 0.53 (95% CI 0.33 to 0.85) in the adjusted model, respectively (Table 2c). Although paternal vegetable intake and the DRS and TRS show negative associations with the KIDScore, the effect diminished when adjusting for maternal age combined with the risk scores of women and failed to reach significance.

## Discussion

In this study, we showed that inadequate maternal vegetable and fruit intake, as well as paternal smoking during the periconception period, are associated with the quality and developmental morphokinetics of pre-implantation embryos as outcome of implantation potential. Inadequate periconceptional maternal vegetable intake was negatively associated with the quality of resulting ICSI embryos. Moreover, the effect size was more than doubled in women with a BMI > 25 kg/m^2^. In men, we observed that smoking was negatively associated with embryo quality as measured by the KIDScore. Importantly, our results highlight that a majority of subfertile couples undergoing ICSI treatment are not adherent to a healthy diet and lifestyle in the months preceding oocyte retrieval, semen collection, and subsequent fertilization. Despite the fact that many couples score low on a healthy diet and lifestyle, we did not observe associations between these factors and the proportion of underdevelopment of embryos, which were discarded.

Our results are in line with the study of Braga et al.*,* which showed that periconceptional intake of fruits and vegetables significantly improved pre-implantation embryo quality after ICSI treatment, whereas fruit intake was also positively associated with blastocyst formation [31]. No effects of fruit and vegetable intake were seen regarding clinical ongoing pregnancy rates, which might be attributed to the fact that this study is underpowered for this outcome. However, Braga et al. did not use the KIDScore as outcome parameter, but only performed conventional, static, embryo morphology assessment on day 3. They showed an effect of maternal smoking and alcohol use, although the proportion of women that smoked in their study was not mentioned. The absence of a clear effect of alcohol consumption on pre-implantation embryo quality, could be explained by the fact that in our cohort only a few women reported using alcohol. Moreover, as few women reported smoking and nearly all women used folic acid in our study population, statistical analysis on smoking and folic acid use was not meaningful.

The correlation between a maternal healthy diet and periconceptional outcomes has been well established [3]. Vujkovic et al. showed that adherence to a healthy “Mediterranean” dietary pattern, which consists of high intake of vegetable oils, vegetables, fish, and legumes and low intake of snacks, is associated with an increased chance (odds ratio: 1.4) of pregnancy in couples undergoing an IVF/ICSI treatment [32]. Inversely, an unhealthy diet characterized by low levels of folate, zinc, and antioxidants is associated with a decreased chance of pregnancy [33]. Importantly, a Western diet with high intakes of pizza and potatoes and low intake of fruit was associated with a nearly two-fold increase in developing a congenital cleft lip [34]. In line with these findings, Oostingh et al. recently reported in a review that inadequate maternal nutrition is associated with lower fecundity and that an optimal maternal vitamin status is associated with decreased risk of first-trimester miscarriage [3]. The positive associations in our study between maternal vegetables, fruits, paternal smoking, and pre-implantation embryo quality indicate that both maternal and paternal factors already influence initiation of embryo potential and development capacity directly postconceptionally. These effects could be explained by the fact that fruits and vegetables are rich in exogenous antioxidants, such as vitamin C and vitamin E, and also in elements with antioxidant properties such as folate and zinc. Antioxidants can provide protection of DNA against oxidative stress caused by reactive oxygen species (ROS), which are produced as a byproduct in the process of aerobic metabolism necessary for normal physiological function of DNA replication. However, excessive oxidative stress can result in DNA damage (single and double strand breaks and chromosomal rearrangements) and in sperm also leads to decreased mitochondrial function necessary for seminal propulsion with resulting impaired motility [35]. Interestingly, our results are only present in the overweight/obese group as compared to the normal weight group. Overweight and obesity can be considered a chronic inflammatory state, with fat cells releasing inflammatory factors and thereby inducing a pro-inflammatory state and oxidative stress [36]. Possibly obesity and inadequate fruit and vegetable intake work synergistically and the combination of both induces too much oxidative stress during oogenesis, which results in embryos with less optimal developmental potential.

Paternal smoking and high BMI can have a detrimental effect on all semen parameters like volume, density, concentration and morphology [37, 38], which are linked to reproductive success. It is known that smoking can increase DNA damage and aneuploidies present in sperm and is associated with, or even can be the cause of, congenital malformations [39]. Recent literature shows that DNA damage and sperm epigenetic information are also transferred to the embryo [40, 41]. Our results point to an effect of paternal smoking on sperm that is directly carried over to the pre-implantation embryo as it is associated with less optimal early development and hence lower embryo quality based on the scored morphokinetic parameters. Although we find associations regarding paternal vegetable intake and the embryo KIDScore with similar effect estimates and odds ratios as for the women, these associations disappear when corrected for maternal total risk scores. Since similar odds ratios were seen for men and women, this could possibly be attributed to a power problem. We decided to correct for the maternal risk scores, since we assume that the corresponding effect estimates are due to high correlation between couples regarding their eating habits and lifestyle factors. We are therefore unable to determine who of both does contribute most to the detrimental effects on early developments, the woman or the man. However, since the number of participating women in the current study is far larger than men, we decided to adjust for maternal risk scores in the male analysis. In larger cohorts, we want to argue for the interpretation of the paternal results without corrections for their female partners.

A strength of this study is the use of the validated mHealth Smarter pregnancy program using the dietary risk score. Another strength is the use of the KIDScore to evaluate embryo quality, which is generally used and easily applicable. A KIDScore of 5 is associated with high implantation rates, where a low KIDScore of 1 is associated with low implantation rates. Despite its general and easy applicability, the predictive capability of the KIDScore on implantation has an AUC of 0.65, which could be classified as a fair predictor. In addition, we have to consider for inference of our data that developmental kinetics at the cleavage stages are also reflective of genetic integrity and blastocyst quality. Our study was conducted on embryos cultured until day 3 in a time period that culturing until day 3 was routine clinical practice in most hospitals and also in our hospital. From 2019 onwards, we are culturing the embryos until the blastocyst stage (day 5). Therefore, future research should also investigate the associations between periconceptional parental nutrition and lifestyle factors and the outcome after culturing until day 5, i.e., blastocyst quality, pregnancy rates, and outcomes. In our study we did not investigate the association between nutritional and lifestyle scores on clinical pregnancy rate (detectable embryonic heartbeat), which should be done in larger cohorts. Another strength is that using pre-implantation embryo development in vitro allows studying early development outside of the maternal body, allowing contribution and identification of paternal as well as maternal factors from both parental gametes. The uterine environment makes identification of paternal factors impossible, since factors of the uterine environment could bypass and dampen paternal effects, while following early development and implantation in utero is still technically impossible. Importantly, we show that paternal lifestyle significantly impacts on embryo developmental kinetics during the cleavage stages. Since the study population consists of subfertile couples visiting a tertiary university-based hospital, although it does not mean all couples are in need of tertiary referral or care, the results cannot be automatically extrapolated to the general fertile population and this might have consequences for the external validity of our study. All couples underwent ICSI treatment, so little information is available on IVF and female factor subfertility. However, studying the effects of pre-implantation embryo development can only be performed in fertility centres which have availability of time-lapse imaging. Bias to our results cannot be excluded, because only 36% of the male partners were willing to participate. This study revealed the magnitude of effect sizes which will help us to better calculate the sample size for future larger studies or randomized controlled trials.

We realized that specific subfertility-related diseases, such as endometriosis and polycystic ovary syndrome (PCOS), are also associated with oocyte and embryo quality. Endometriosis negatively affects the oocyte quality, which could be caused by increased oxidative stress [42]. PCOS is associated with decreased embryo quality, defined as slower development during the cleavage stage [43]. Although our study population consisted of 113 women, it was statistically not possible to correct or stratify for these possible confounders. Future studies in larger populations should take these confounders into consideration.

This study showed that both periconceptional maternal and paternal lifestyle and nutritional factors already have an impact on pre-implantation embryo quality based on morphokinetic evaluation after time-lapse culture. As more than 80% of the reproductive population has one or more inadequate nutrition and lifestyle behaviors, studying and modifying the associations by evidence-based interventions is becoming increasingly important. Moreover, the live birth rate per started IVF/ICSI cycle has remained stable at around 30% per cycle for the last decade. With the increase of subfertility in the Western world, it is essential to determine how to stop the increasing subfertility numbers, improve ART chances and prevent and overcome subfertility causes. Therefore, we conclude that identifying modifiable risk factors, as a first step in the behavioral change pathway to stimulate awareness, can have already a clinical impact by increasing the chance of a healthy pregnancy and having a live-born baby.

## Electronic Supplementary Material

ESM 1 Odds ratios and confidence intervals of the associations between the risk scores for vegetable intake, fruit intake and the dietary risk score and the KIDScore in the female population depicted for the complete group and women with BMI ≥25 kg/m^2^ and BMI<25 kg/m^2^. (PNG 172 kb)

High Resolution Image (TIF 19247 kb)

ESM 2 (DOCX 49 kb)
