# Supplementary figures and images for: Preconceptional Maternal Vegetable Intake and Paternal Smoking Are Associated with Pre-implantation Embryo Quality
Source: Reprod Sci. 2020 Jun 15;27(11):2018–28. doi: 10.1007/s43032-020-00220-8 (PMC7522074; doi:10.1007/s43032-020-00220-8)

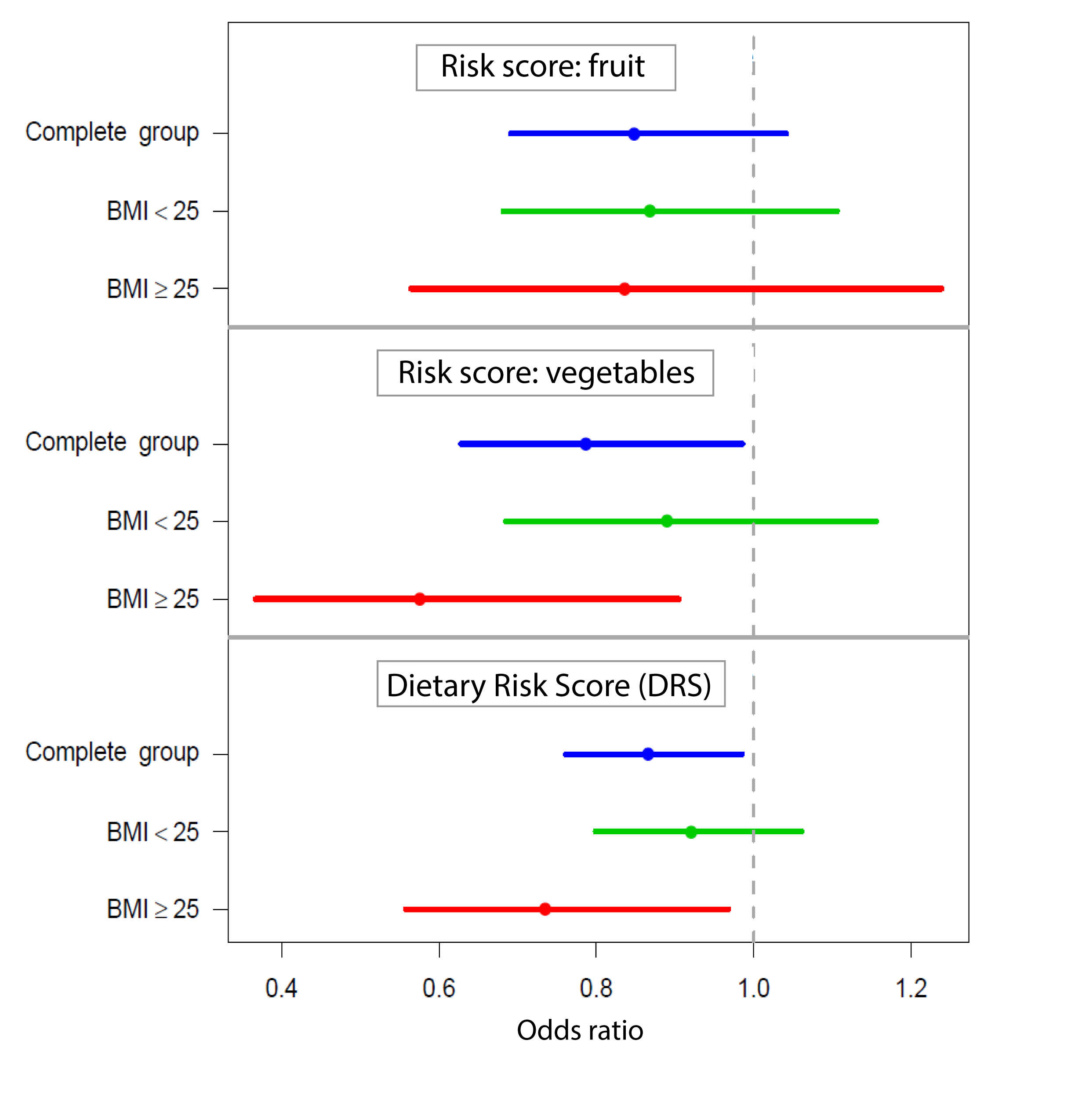

Supplement: Supplementary file 1 — Odds ratios and confidence intervals of the associations between the risk scores for vegetable intake, fruit intake and the dietary risk score and the KIDScore in the female population depicted for the complete group and women with BMI ≥25 kg/m2 and BMI<25 kg/m2. (PNG 172 kb) [file 43032_2020_220_Fig3_ESM.png]

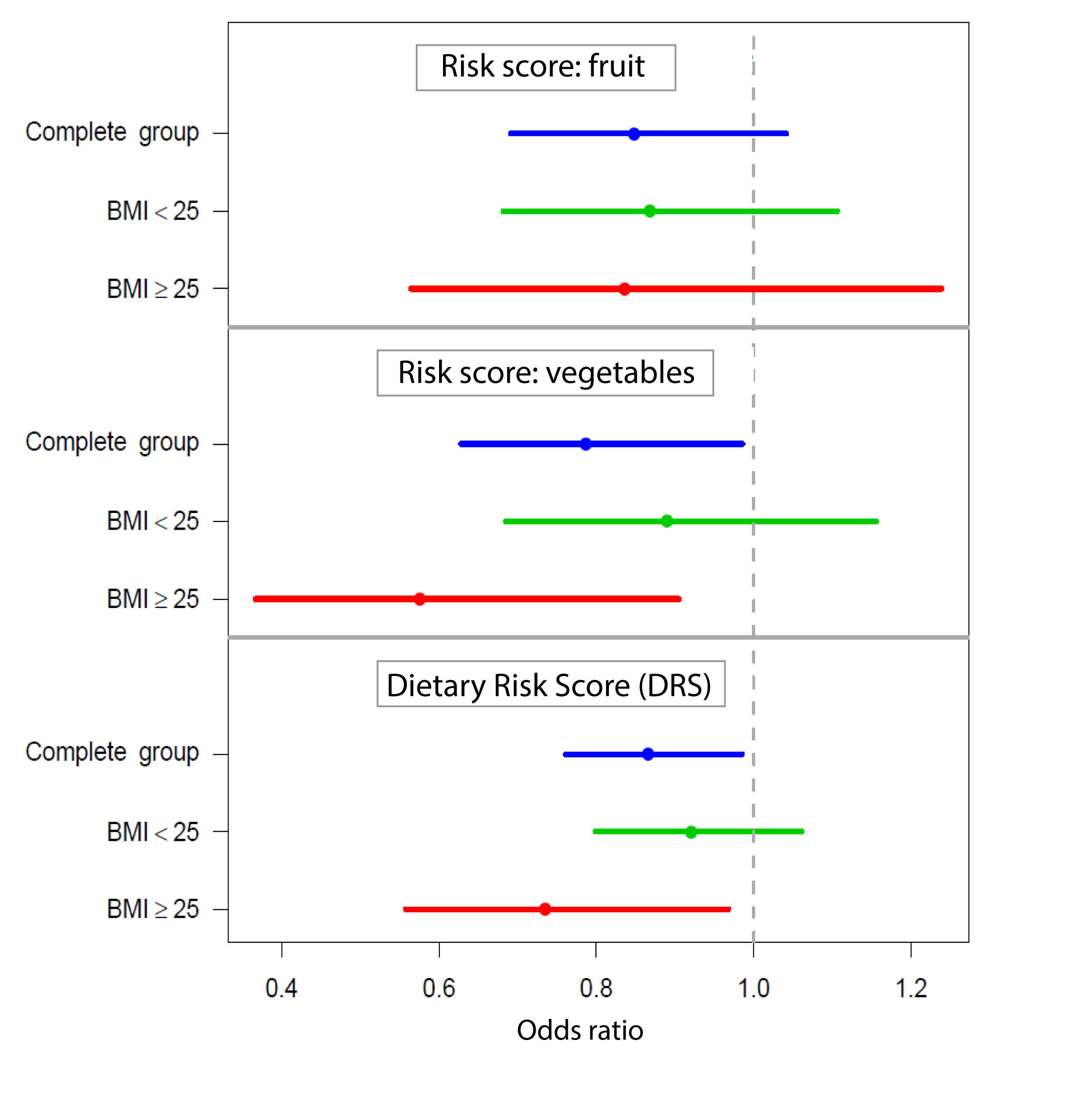

Supplement: Supplementary file 2 — High Resolution Image (TIF 19247 kb) [file 43032_2020_220_MOESM1_ESM.tif]
