# Supplementary material for: Preconceptional Maternal Vegetable Intake and Paternal Smoking Are Associated with Pre-implantation Embryo Quality
Source: Reprod Sci. 2020 Jun 15;27(11):2018–28. doi: 10.1007/s43032-020-00220-8 (PMC7522074; doi:10.1007/s43032-020-00220-8)
Supplement: Supplementary file 3 — (DOCX 49 kb) [file 43032_2020_220_MOESM2_ESM.docx]

**Supplemental table 1.** Effect estimates and odds ratios from the proportional odds model for the nutrition and lifestyle risk scores on the KIDScore for normal weight women only.

|  | **Crude** | | | **Adjusted** | | |
| --- | --- | --- | --- | --- | --- | --- |
|  | Effect estimate | Odds ratio (95 % CI) | P-value | Effect estimate | Odds ratio (95 % CI) | P-value |
| Total Risk Score | -0,06 | 0,94 (0.82 to 1.08) | 0.39 | -0,06 | 0,94 (0.82 to 1.08) | 0.39 |
| Dietary Risk Score | -0,08 | 0,92 (0.80 to 1.06) | 0.25 | -0,09 | 0,92 (0.80 to 1.06) | 0.24 |
| Lifestyle Risk Score | 0,15 | 1,16 (0.78 to 1.71) | 0.47 | 0,16 | 1,18 (0.79 to 1.74) | 0.42 |
| Vegetable intake | -0,12 | 0,89 (0.69 to 1.15) | 0.38 | -0,12 | 0,89 (0.68 to 1.17) | 0.39 |
| Fruit intake | -0,14 | 0,87 (0.68 to 1.11) | 0.25 | -0,14 | 0,87 (0.68 to 1.10) | 0.24 |
| Folic acid supplement use | n/a |  |  | n/a |  |  |
| Alcohol use | 0,15 | 1,16 (0.78 to 1.71) | 0.47 | 0,16 | 1,18 (0.79 to 1.74) | 0.42 |
| Smoking | n/a |  |  | n/a |  |  |

Model 1: crude model

Model 2: Model 1 + adjusted for maternal age and the corresponding risk score from the women

**Supplemental table 2.** Effect estimates and odds ratios from the generalized linear mixed model for the association between the nutrition and lifestyle risk scores on the proportion of discarded embryos.

|  | **Crude** | | | **Adjusted** | | |
| --- | --- | --- | --- | --- | --- | --- |
|  | Effect estimate | Odds ratio  (95 % CI) | P-value | Effect estimate | Odds ratio  (95 % CI) | P-value |
| Total Risk Score | -0.02 | 0.98 (0.88 to 1.08) | 0.68 | 0.10 | 1.11 (0.92 to 1.33) | 0.53 |
| Dietary Risk Score | 0.01 | 1.01 (0.91 to 1.13) | 0.83 | 0.07 | 1.07 (0.89 to 1.29) | 0.93 |
| Lifestyle Risk Score | -0.28 | 0.75 (0.56 to 1.02) | 0.06 | 0.27 | 1.31 (0.77 to 2.20) | 0.10 |
| Vegetable intake | -0.01 | 1.00 (0.82 to 1.21) | 0.96 | -0.08 | 0.92 (0.64 to 1.33) | 0.69 |
| Fruit intake | 0.06 | 1.07 (0.89 to 1.28) | 0.49 | 0.26 | 1.29 (0.96 to 1.75) | 0.60 |
| Folic acid supplement use | n/a |  |  | n/a |  |  |
| Alcohol use | -0.28 | 0.76 (0.56 to 1.02) | 0.06 | 0.27 | 1.31 (0.77 to 2.20) | 0.10 |
| Smoking | n/a |  |  | n/a |  |  |

Model 1: crude model

Model 2: Model 1 + adjusted for maternal age
